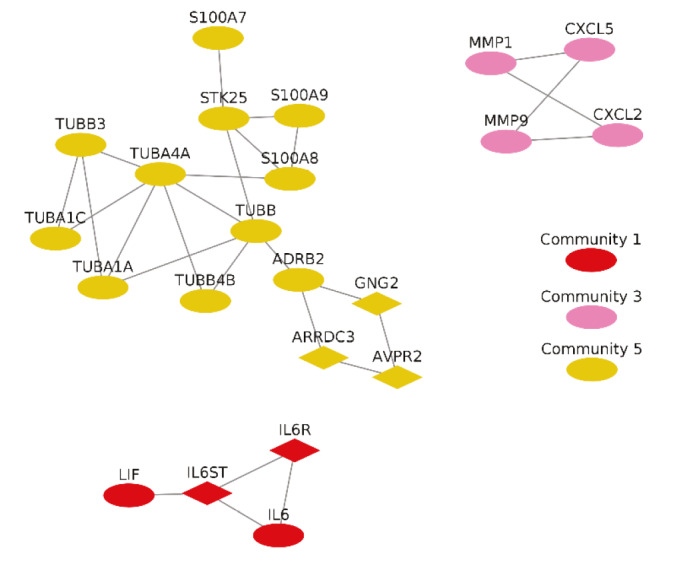# Integration of transcriptomic profile of SARS-CoV-2 infected normal human bronchial epithelial cells with metabolic and protein-protein interaction networks

**DOI:** 10.3906/biy-2005-115

**Published:** 2020-06-21

**Authors:** Hamza Umut KARAKURT, Pınar PİR

**Affiliations:** 1 Department of Bioengineering, Faculty of Engineering, Gebze Technical University, Kocaeli Turkey; 2 Idea Technology Solutions, İstanbul Turkey

**Keywords:** SARS-CoV-2, bioinformatics, transcriptome, metabolome, biological networks, data integration

## Abstract

A novel coronavirus (SARS-CoV-2, formerly known as nCoV-2019) that causes an acute respiratory disease has emerged in Wuhan, China and spread globally in early 2020. On January the 30th, the World Health Organization (WHO) declared spread of this virus as an epidemic and a public health emergency. With its highly contagious characteristic and long incubation time, confinement of SARS-CoV-2 requires drastic lock-down measures to be taken and therefore early diagnosis is crucial. We analysed transcriptome of SARS-CoV-2 infected human lung epithelial cells, compared it with mock-infected cells, used network-based reporter metabolite approach and integrated the transcriptome data with protein-protein interaction network to elucidate the early cellular response. Significantly affected metabolites have the potential to be used in diagnostics while pathways of protein clusters have the potential to be used as targets for supportive or novel therapeutic approaches. Our results are in accordance with the literature on response of IL6 family of cytokines and their importance, in addition, we find that matrix metalloproteinase 2 (MMP2) and matrix metalloproteinase 9 (MMP9) with keratan sulfate synthesis pathway may play a key role in the infection. We hypothesize that MMP9 inhibitors have potential to prevent "cytokine storm" in severely affected patients.

## 1. Introduction

In December 2019, a group of pneumonia patients have been identified to be infected with a novel coronavirus, SARS-CoV-2. Since then, SARS-CoV-2 has spread around the globe and the World Health Organization (WHO) declared COVID-19 as a public health emergency and an epidemicc (Guo et al., 2020). As of May 29th, 2020, the number of confirmed cases approaches 6 million and death toll is about 364 thousand according to WHO. The other 2 strains of β-coronaviruses, severe acute respiratory syndrome coronavirus (SARS-CoV) and Middle East respiratory syndrome coronavirus (MERS-CoV) have zoonotic origins. Early patients in Wuhan, China were also epidemiologically linked to a seafood and wet animal wholesale market (Zhu et al., 2020).

The common symptoms of SARS-CoV-2 infections are fever, cough and dyspnea according to early clinical studies on confirmed patients. Global efforts towards developing vaccine is in progress, however, according to WHO, it is anticipated to be available in 18 months. Currently, supportive antiviral agents, therapeutics such as a malaria drug, chloroquine (Wang et al., 2020) and respiratory support systems are being used for treatment of the patients (Guo et al., 2020; Jiang et al., 2020).

SARS-CoV-2 is genetically different from SARS-CoV, and phylogenetic analyses suggested that it may be originated from bats. Structural analyses indicated that receptor protein for virus binding is angiotensin-converting enzyme 2 receptor (ACE2) in humans (Lu et al., 2020). Although lung epithelial cells are considered to be the major host of SARS-CoV-2, single-cell RNA-seq analyses identified potential host cell types in humans and curiously lung epithelial cells had relatively low expression levels of ACE2 (Zou et al., 2020).

In the response to pathogens, signals transmitted via cytokines recruit immune cells to the site of infection. In a positive feedback loop, cytokines activate immune cells and stimulate them to produce more cytokines. However, in some cases, the response is not moderated, and excessive numbers of immune cells are recruited in the microenvironment. In turn, extreme rally of immune cells can cause local tissue and organ damage. Interferons, interleukins, chemokines, colony-stimulating factors (CSFs) and tumour necrosis factors (TNFs) are cytokine families that are associated with cytokine storm (Tisoncik et al., 2012). The term “cytokine storm” was coined in early 2000s to describe extreme elevation of inflammatory elements and was observed in patients infected with a diverse set of viruses including SARS-CoV and Epstein-Barr viruses. Cytokine storm is excessive expression of cytokines in response to an external signal, and it was reported that patients with severe SARS-CoV-2 infection also show symptoms of cytokine storm (Ye et al., 2020). Cytokine storm may lead to complications such as systemic inflammation, multi-organ failure and if not treated, loss of the patient (Tisoncik et al., 2012). 

The response of lung epithelial cells is crucial in immune defence against SARS-CoV-2. The response in signalling pathways, gene expression, protein levels and metabolic profiles are regulated as a result of interactions in multilayer biological networks, hence a holistic view of the cellular response can be elucidated by an integrated approach. Here, we provide an analysis of transcriptional response after 24 h of infection, further, we integrated transcriptome profile with metabolic and protein-protein interaction networks to reveal multilayer mechanistic details of the SARS-CoV-2 infection in NHBE cells. Our analysis identified the genes, pathways, metabolites and protein interactions that are markers of infected state. The identified metabolites, and proteins and their interactions have the potential to be used as biomarkers and drug targets. 

## 2. Material and methods

A public RNA-seq data (Blanco-Melo et al., 2020) (GSE147507), from NCBI Gene Expression Omnibus (GEO) (Barrett et al., 2013) was used in this study. Raw read files (in fastq format) were downloaded from NCBI Sequence Read Archive (SRA) (National Centre for Biotechnology Information, 2015) and aligned with Kallisto pseudo-aligner (Bray et al., 2016) using Ensembl Homo Sapiens Transcriptome v96. Differential expression (DE) analysis is done using DESeq2 (Love et al., 2014) and visualized using enhanced volcano (Blighe, 2019) package. Enrichment analyses are done using clusterProfiler package (Yu et al., 2012) based on pathways and terms from Kyoto encyclopedia of genes and genomes (KEGG) and gene ontology biological process (GO-BP) databases, respectively. DE analysis results (P-values) are imported to Matlab 2020a for reporter metabolite (RMA) (Patil and Nielsen, 2005) and reporter pathway (RPA) (Çakır, 2015) analyses. For RMA and RPA, the metabolic network of bronchus respiratory epithelial cell metabolic model (based on Recon2) (Thiele et al., 2013) is used.

Significantly changed genes and their log2 fold change (log2FC) values are integrated with protein-protein interaction (PPI) network using Prize-collecting Steiner forest algorithm in PCSF package (Akhmedov et al., 2017) and STRING (Szklarczyk et al., 2015) human PPI network. Integrated network is divided into communities with maximum modularity using Louvain community detection (Blondel et al., 2008) algorithm in igraph (Csardi and Nepusz, 2006) package for R. 

For identification of potentially active clusters in the protein-protein interaction network, ClusterONE (Nepusz et al., 2012) algorithm is used in Cytoscape. Clusters with the P-value lower than 0.1 and with more than 4 nodes were considered for further analysis. All networks are visualized in Cytoscape desktop application (Shannon et al., 2003).

## 3. Results

### 3.1. Differential expression analysis results

**Figure 1:**
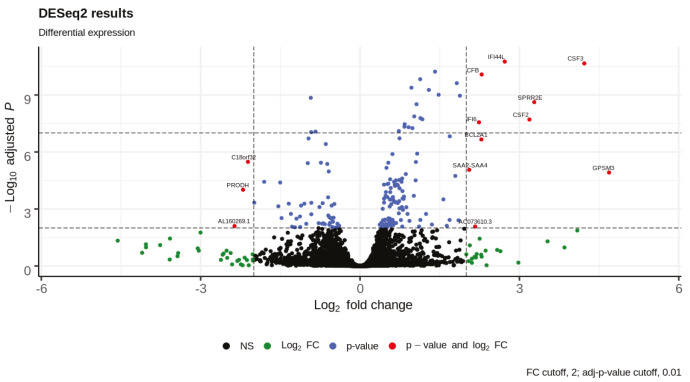
Volcano plot of differential expression analysis results.

**Figure 2:**
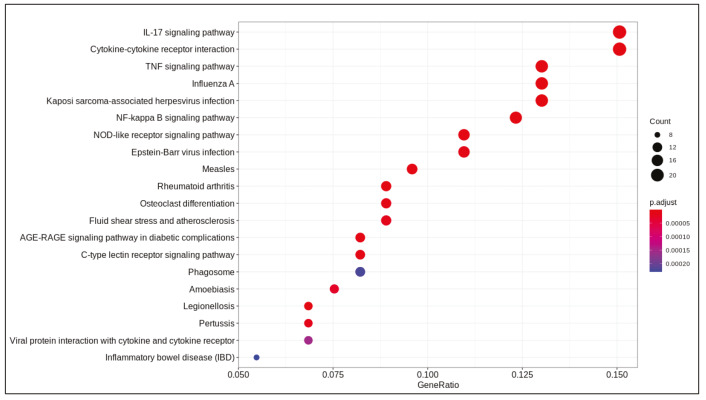
Dotplot of enrichment analysis results (KEGG).

**Figure 3:**
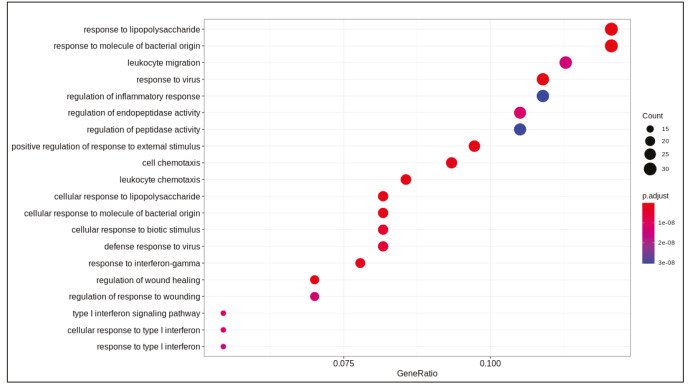
Dotplot of enrichment analysis results (GOBP).

DE analysis identified 217 significantly changed (adjusted P-value < 0.01) genes (165 upregulated and 51 downregulated) (Supplementary Table 1) in the response to SARS-CoV-2 infection (Figure 1). Of note, the gene encoding the potential virus entry protein (angiotensin-converting enzyme 2 gene, ACE2) did not change its expression in NHBE cells in response to SARS-CoV-2, indeed, expression values of ACE2 in all samples are similar to each other (Supplementary Figure 1). Enrichment analysis of the significantly changed genes in KEGG pathways indicated that these genes are associated with IL-17 signalling pathway, cytokine-cytokine receptor interaction, TNF signalling pathway, NF-kappa B and NOD-like receptor signalling (Figure 2). Analyses on GO-BP terms indicated that inflammatory response, leukocyte migration and response to lipopolysaccharide are the most enriched terms (Figure 3). Extended enrichment analyses for both upregulated and downregulated genes are shown in Supplementary Figures 2–5.

Differential gene expression results indicated alterations in viral infection response pathways and signalling pathways. Cytokine-cytokine receptor activation, IL-17 signalling (Tufan and Avanoğlu Güler, 2020) and TNF signalling (Zhang et al., 2020) are pathways known to response to SARS-CoV-2 infection. Previously, NF-kappa B signalling pathway activation is found to be associated with the immune response activation in the SARS coronavirus infection (Liao et al., 2005). Pathways associated with infection of other viruses such as influenza A, Epstein-Barr, measles, and nonviral disease-associated pathways such as rheumatoid arthritis, AGE-RAGE signalling pathway in diabetic complications were also enriched in our analyses.

Transcriptional response of these pathways in NHBE to SARS-CoV-2 infection were analysed previously together with response in cancer cell lines, ferrets and serum samples (Blanco-Melo et al., 2020). Despite lack of a robust IFN-I and -III response, high levels of a subset of cytokines such as CCL20, CXCL1, CXCL3, CXCL5, CXCL6, CXCL2, CXCL16, IL-6, IL1β and TNF was detected. It was suggested that treatments for COVID-19 should be based on controlling the inflammation rather than IFN response as SARS-CoV-2 response is imbalanced with regard to controlling virus replication versus activation of the adaptive immune system. In a further analysis of response in NHBE cells to SARS-CoV-2 infection, feedback loops leading to cytokine expression was mapped on signalling pathways to elucidate the mechanism of response and IL1βand TNF were found to be central in crosstalk of IL-17, TNF and NFκB pathways in cytokine production.

### 3.2. Reporter metabolite and pathway analysis

Comparison of gene transcription across conditions provides an overview of the transcriptional response, whereas mapping the transcriptome on biological networks provides insights on potential impact on other layers of cellular regulation. We aimed to investigate the potential impact of SARS-CoV-2 infection on metabolism and metabolites by mapping the significantly changed genes on metabolic networks. Our analysis is based on the assumption that if the level of a gene encoding the enzyme of a metabolic reaction has significantly changed, this transcriptional change will impose a change in the reaction flux, therefore the relevant metabolite levels will also be affected. Such metabolic alterations in the response to SARS-CoV-2 infection have the potential to be used as biomarkers in noninvasive test methods such as analysis of blood samples. 

**Figure 4:**
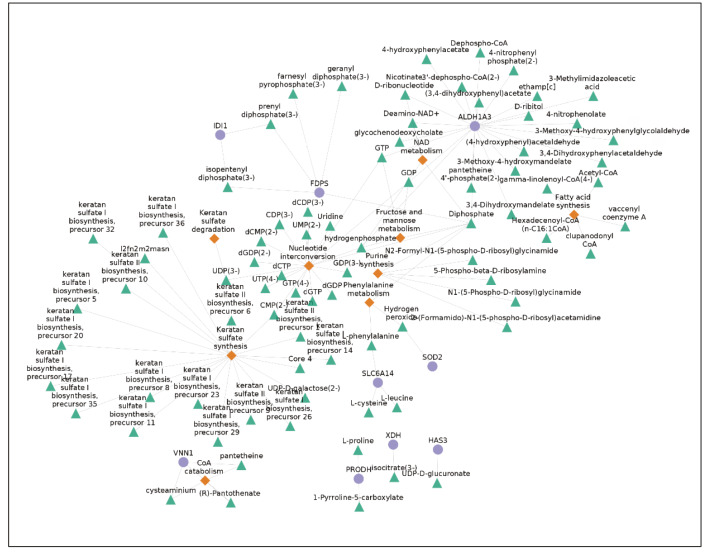
Reporter metabolites and pathways (blue circles; genes, green triangles; metabolite, orange diamonds; pathways).

Reporter metabolite analysis (RMA) and reporter pathway analysis (RPA) identified 106 significantly affected metabolites and 11 significantly affected pathways (P-value < 0.05) in response to infection. The network of significantly changed metabolites and pathways along with differentially expressed gene connections is shown in Figure 4. Only significantly altered genes and metabolites on significantly altered pathways are shown for clarity, which means that only connected nodes (degree equal or more than 1) are shown, and significantly altered genes or metabolites from nonsignificant pathways are omitted. All results are shown in Supplementary Tables 2 and 3.

Keratan sulfate synthesis is the most significantly affected pathway with the highest number of connected metabolites. Keratan sulfates are a subclass of glycosaminoglycans (GAG), class of complex-linear polysaccharides which interact with various molecules (Aquino and Park, 2016) and have been shown to bind a wide range of pathogens including viruses (Jinno and Park, 2015; Chandra et al., 2019). Our analysis indicated that after 24 h of infection, the synthesis of keratan sulfate is altered in NHBE cells. ALDH1A3, an enzyme from aldehyde dehydrogenase 1 family is significantly upregulated in NHBE cells and is the gene with highest degree in the network. There is not any experimental evidence about the regulation of this gene in viral infections but it is shown that ALDH1A3 regulates 2 matricellular proteins (TNC1 and ESM1) in vascular smooth muscle cell proliferation (Xie et al., 2019). Enrichment analyses of downregulated genes with GO-BP terms indicated that extracellular matrix organization is downregulated with the viral infection. The connection between extracellular matrix regulation and significantly altered metabolites in RMA results indicate that ALDH1A3 may take part in regulation of numerous biological subsystems such as extracellular matrix, NAD metabolism and fatty acid metabolism in the response. As fatty acids can modulate the extracellular matrix, ALDH1A3 may mediate extracellular matrix alteration response with its dual metabolic and regulatory functions. 

### 3.3. Integration with protein-protein interaction network

To extend our analysis to another layer of cellular regulation, we mapped the transcriptome on protein-protein interaction (PPI) network. PPI networks provide a global view of cooperation between proteins in cellular processes, therefore subnetworks of overexpressed proteins may identify how the response to virus is orchestrated by the cell. Thus, we attempted integration of transcriptome data to protein-protein interaction networks with prize-collecting Steiner forest (PCSF) algorithm. PCSF method allows inclusion of non-responding genes (‘Steiner nodes’) to the subnetworks, therefore non-responding genes that connects the differentially expressed genes can also be identified. 

**Figure 5:**
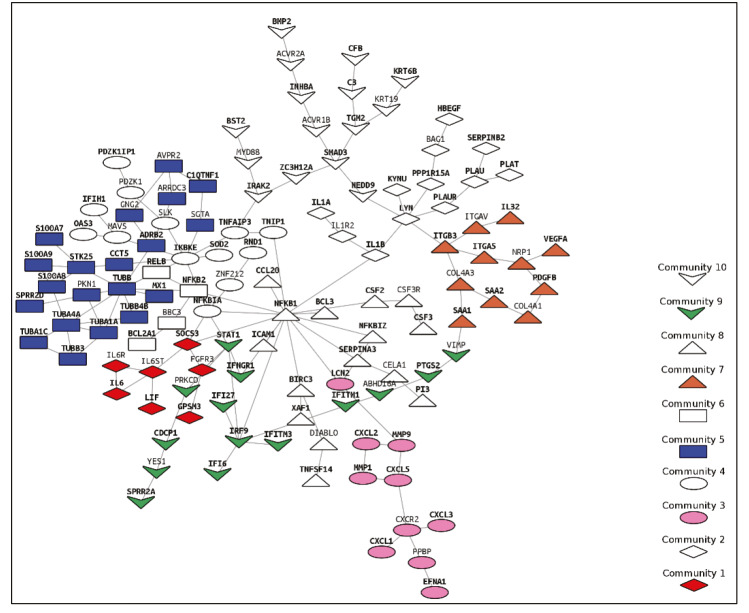
PPI subnetwork obtained via PCSF with communities (Louvain algorithm) (bold labelled nodes; terminal nodes).

PCSF algorithm extracted an active PPI subnetwork of 131 nodes (32 Steiner and 89 terminal nodes) and 143 edges in NHBE cells in response to virus infection. Louvain community detection algorithm identified 10 communities within the active PPI subnetwork (Figure 5). Genes encoding the proteins in each community are analysed for functional enrichment (Supplementary Figures 6 and 7; Supplementary Tables 4 and 5). Further, PPI network with communities are analyzed with ClusterONE to identify potentially functional PPI modules (clusters) in the network (Supplementary Figure 8). Each cluster is separately analysed for functional enrichment (Supplementary Figures 9 and 10).

**Figure 6:**
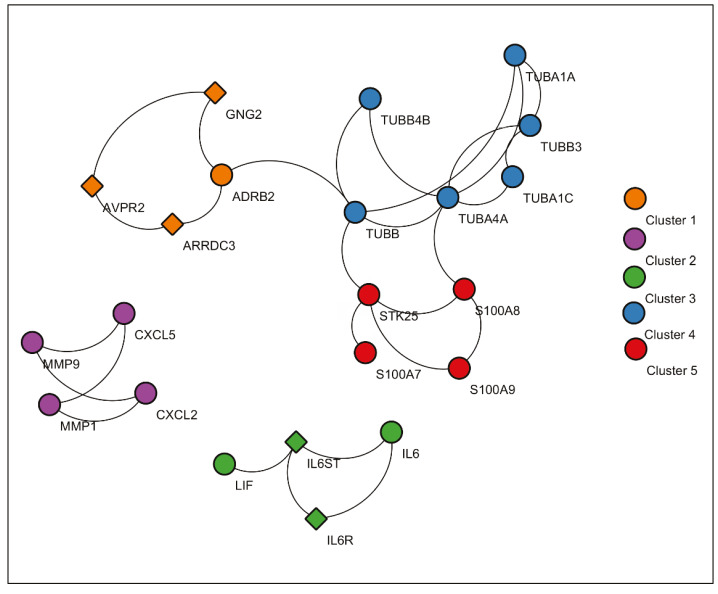
Significant protein clusters (P-value < 0.1).

Significant clusters identified via ClusterONE is shown in Figure 6. Three of 5 significant clusters comes from the 1 large community with 20 nodes and 27 edges (Community 5) while other 2 comes from Communities 1 and 3 (Supplementary Figure 11). Enrichment results of this community show significant association with metal-ion sequestering, G2/M cycle, organelle localization and phagosome. Neurodegeneration associated terms (Huntington’s disease and Alzheimer’s disease) are also found in the results. These results indicate that a wide range of processes are affected from the infection, or perhaps multifunctional genes which take part in above mentioned processes also respond to viral infections.

Proteins in Communities 1, 3, and 4 are enriched in signalling pathways and biological processes known to respond to virus infections (Supplementary Figures 6 and 7) which are potential drug target pathways. In addition to community detection, clustering of PPI also highlights functional interactions. Interleukin-6 (IL-6), Interleukin-6 receptor (IL6R), and Interleukin-6 signal (IL6ST) constitute Cluster 3 together with LIF Interleukin 6 Family Cytokine (also known as leukemia inhibitory factor, LIF). Previously it was reported that LIF protects the lung during respiratory syncytial viral infection (Foronjy et al., 2014). Similar to IL-6 levels being a predictive factor in respiratory failure (Herold et al., 2020), LIF levels may indicate the severity of the infection in patients. C-X-C motif chemokine ligands 2 and 5 (CXCL2 and CXCL5) form the Cluster 2 with matrix metalloproteinases 1 and 9 (MMP1 and MMP9). Previously, it was shown that in cells infected with respiratory syncytial virus (RSV) and human immunodeficiency virus-1 (HIV-1), levels of CXCL2 and CXCL5 increase (Miller et al., 2004; Guha et al., 2015). 

In vitro studies in human astrocytic and microglial cells with human coronavirus showed an increase in the levels of MMP2 and MMP9 in infection. In addition, IL-6 and TNF-α are inducers of MMP expression (Marten and Zhou, 2005). In another study, MMP9 levels were increased with RSV infection in the mice lungs and knockout mutants had increased RSV-induced airway hyperresponsiveness and reduced clearance (Dabo et al., 2015). NHBE transcriptome data analysed in this study is in accordance with these observations. Further, PPI integrated with transcriptome indicated that MMP2 and MMP9 activity are connected to CXCL2 and CXCL5 as mentioned above and this relationship may have a significant function in SARS-CoV-2 infection. 

MMP family regulates cytokine processing, leucocyte migration and matrix remodelling. These proteins were found to be associated with viral, bacterial, fungal and parasitic infections and it was reported that extreme activity of MMPs may cause host morbidity (Elkington et al., 2005). Significant upregulation in expression of matrix metalloproteinase 9 (MMP9) in NHBE cells indicates that drugs which target MMP9 have potential uses in SARS-CoV-2 infection. 

## 4. Discussion

We presented a multilayer analysis of cellular response to SARS-CoV-2 infection, that spans from gene regulation to protein-protein interaction and metabolic networks. Nonviral diseases that appear on our list of enriched pathways indicate that virus response and gene dysregulation in other diseases are intertwined, partly explaining the success of drug repositioning currently being applied in treatment of COVID-19 patients. JAK-STAT inhibitors such as baricitinib, fedratinib, and ruxolitinib are approved drugs for rheumatoid arthritis treatment and currently used for the treatment of COVID-19 patients through the suppression of elevated cytokine levels (Stebbing et al., 2020). Hydroxychloroquine, a known antimalaria drug and has potential use in rheumatoid arthritis is used in patients (Colson et al., 2020) with promising results. Interactions between advanced glycation end product (AGE) and its receptor (RAGE) have a role in the pathogenesis of pneumonia (Van Zoelen et al., 2011) and have potential use as biomarker or treatment method in inflammatory diseases (Hudson and Lippman, 2018). To best of our knowledge, any direct evidence about SARS-CoV-2 and RAGE receptor relation is not yet reported in the literature. This pathway also shares ligands with Toll-like receptor family (Chavakis et al., 2004). In addition to these pathways and genes, enriched GO-BP terms indicated that significantly changed genes are highly associated with response to lipopolysaccharide. Previous studies indicated that AGEs and lipopolysaccharides stimulate interleukin‑6 (IL-6) secretion via the RAGE/TLR4‑NF‑κB‑ROS pathways in mice (Ohtsu et al., 2017). Levels of IL-6 found to have potential to predict respiratory failure in patients (Herold et al., 2020). Thus far, specific signalling pathway associated drugs are used for SARS-CoV-2 patients to suppress the symptoms. This complex network structure between signalling pathways indicated that RAGE receptor targeting drugs have the potential to be used in SARS-CoV-2 patients to suppress symptoms.

Previously, it was reported that deficiency of matrix metalloproteinase 9 (MMP9) reduce severity of IAV infection in mice. Further, increased levels of host proteinases including trypsin, which can activate pro-MMP9, was associated with cytokine storm (Rojas-Quintero et al., 2018). Processing of CXCL5 by MMP2 and MMP9 promotes neutrophil recruitment demonstrating the regulatory effect of matrix metalloproteinases on chemokines (Song et al., 2013). In our PPI clusters, the neutrophil chemoattractant chemokines CXCL2 and CXCL5 clustered together with MMP2 and MMP9, indicating coexpression and a functional interaction between these proteins. Taken together, in SARS-CoV-2 infection, increased MMP9 may regulate CXCL protein family. Early recruitment of neutrophils may lead to the cytokine storm observed in SARS-CoV-2 infection. Therefore, we hypothesize that MMP inhibitors can be used for SARS-CoV-2 infected patients to suppress the cytokine storm. DrugBank (Wishart et al., 2008) lists inhibitors of MMP9 such as marimastat and minocycline, which can be considered for treatment of COVID-19. 

In addition, it was previously reported that MMP activity is regulated via glycosaminoglycans (GAG) (Ruiz-Gómez et al., 2019). Keratan sulfate synthesis along with connected metabolites is 1 of the most statistically significant results of our RMA/RPA analyses. In literature, there is no direct evidence of a connection between MMPs and keratan sulfates but since keratan sulfates are members of sulfated glycosaminoglycan family, their regulation in SARS-CoV-2 infection may influence the activity of MMP2 and MMP9. DrugBank suggests glucosamine, an intermediate metabolite in the synthesis of glycosaminoglycans, as an approved antagonist of MMP9. 

MMP9 was identified to be significant in various diseases, particularly in infectious diseases. Few examples are; MMP9 promotes Zika virus entry into testes (Hui et al., 2020), facilitates West Nile Virus entry to brain (Wang et al., 2008) and expression of MMP9 is enhanced by Epstein-Barr virus (Yoshizaki et al., 1998) and Japanese encephalitis virus (Yang et al., 2012). Also, neutralization of MMP9 increased the oncolytic efficiency of tanapox virus for melanoma (Zhang et al., 2017). As mentioned before, MMP9 knock-out mice compared to wild type mice revealed that MMP9 enhances neutrophil recruitment and cytokine production in RSV infection (Dabo et al., 2015). Effect of MMP9 on neutrophil recruitment, cytokine and chemokine regulation strengthens our hypothesis that inhibition of MMP9 may suppress the cytokine storm in severe Covid-19 patients. Future studies are needed to confirm effectiveness of MMP9 inhibitors on progress of the disease.

## 5. Conclusions

SARS-CoV-2 is likely to bind nasal epithelial cells via ACE2 and migrate down to lungs after the initial replication period. More robust immune response is triggered in the lungs (Mason, 2020). Transcriptome data analyses confirmed relevance of known viral infection pathways and potential drug target pathways which are in the experimental testing phase for COVID-19 response and treatment. To best of our knowledge, any potential drug or study about the roles of AGEs and RAGE receptor in the infection of SARS-CoV-2 is not reported in the literature. Our analysis indicates that a complex interaction between RAGE receptor signalling pathway and other virus-response related pathways such as Toll-like receptor signalling pathway and IL-6 levels may exist. Taken together, advanced glycation end products (AGEs) and their connected pathways have potential to be used as drug targets.

Further, transcriptome data integration with protein-protein interaction network, followed by community detection and clustering of the subnetwork, identified potentially important protein interaction motifs in the infection. For example, rheumatoid arthritis-associated proteins found in the clustering and community detection results may be the targets of rheumatoid arthritis drugs being used in treatment of SARS-CoV-2 patients. Our analyses also indicate that metalloproteinases (MMP1 and MMP9) have potential importance in the SARS-CoV-2 infection and could be tested as drug targets. 

## Informed consent

The authors declare that all reported results are obtained computationally. No novel experimental data is reported in this publication.

## Supporting Information

Supplementary Table 1

**Table:** Differentially expressed genes.

Gene	Log2 fold change	P-value	Adjusted P-values
CXCL8	2.38	1.47E-71	2.16E-67
CCL20	3.04	8.46E-59	6.24E-55
SAA1	2.18	2.55E-48	1.25E-44
TNIP1	1.28	3.15E-39	1.16E-35
SAA2	2.42	4.57E-39	1.35E-35
CXCL1	1.48	2.61E-37	6.40E-34
S100A8	1.91	1.48E-36	3.13E-33
SPRR2D	3.20	2.30E-36	4.23E-33
ICAM1	1.92	3.24E-36	5.32E-33
INHBA	1.78	1.30E-33	1.92E-30
KRT6B	1.51	5.41E-32	7.26E-29
TNFAIP3	1.52	5.49E-31	6.75E-28
C3	1.46	4.91E-30	5.57E-27
IL36G	2.40	8.13E-30	8.56E-27
C15orf48	1.24	9.09E-28	8.94E-25
SOD2	1.56	3.37E-27	3.10E-24
S100A9	1.27	7.94E-27	6.89E-24
CXCL5	3.45	1.68E-25	1.38E-22
IFI27	2.86	7.59E-25	5.89E-22
HBEGF	1.28	2.40E-24	1.77E-21
SLC6A14	1.26	2.61E-24	1.84E-21
SPRR2A	2.00	7.43E-24	4.98E-21
IL6	2.84	2.37E-22	1.52E-19
ZC3H12A	1.54	3.25E-22	2.00E-19
RHCG	1.36	2.28E-21	1.34E-18
IL32	1.18	4.64E-20	2.63E-17
MMP9	2.26	8.19E-20	4.47E-17
SERPINA3	1.48	9.69E-19	5.10E-16
MX1	1.98	1.84E-18	9.34E-16
LIF	1.36	1.09E-17	5.34E-15
OAS2	1.34	1.22E-17	5.82E-15
IRF9	1.37	3.21E-17	1.48E-14
OAS1	1.57	1.15E-16	5.13E-14
CXCL3	2.19	1.44E-16	6.25E-14
PDZK1IP1	2.34	1.62E-16	6.81E-14
CXCL2	1.36	3.03E-15	1.24E-12
IRAK2	1.59	3.28E-15	1.31E-12
PGLYRP4	1.88	6.73E-15	2.61E-12
OAS3	1.17	8.64E-15	3.27E-12
IL1B	0.92	2.64E-14	9.73E-12
IFI44L	2.72	4.88E-14	1.75E-11
CSF3	4.22	6.22E-14	2.18E-11
TNFAIP2	1.41	1.72E-13	5.91E-11
CFB	2.29	2.48E-13	8.32E-11
MAFF	1.13	4.51E-13	1.48E-10
IFITM1	1.82	7.46E-13	2.39E-10
BPGM	0.97	1.32E-12	4.13E-10
HEPHL1	1.30	1.77E-12	5.43E-10
BIRC3	1.48	3.24E-12	9.74E-10
C1QTNF1	1.88	3.71E-12	1.09E-09
KRT15	-0.93	4.87E-12	1.41E-09
SPRR2E	3.28	8.39E-12	2.38E-09
IL1A	1.06	1.10E-11	3.07E-09
IVL	1.02	4.92E-11	1.34E-08
GGT1.1	1.14	6.17E-11	1.65E-08
PLAT	1.17	7.23E-11	1.90E-08
CSF2	3.19	7.56E-11	1.96E-08
IFI6	2.24	1.08E-10	2.74E-08
G0S2	0.84	1.39E-10	3.47E-08
NFKBIZ	0.84	1.91E-10	4.68E-08
NFKB2	0.91	2.06E-10	4.98E-08
NFKBIA	0.99	2.34E-10	5.57E-08
EFNA1	0.73	3.39E-10	7.93E-08
GPNMB	-0.84	3.66E-10	8.43E-08
MXRA5	-0.92	3.99E-10	9.05E-08
XAF1	1.69	6.71E-10	1.50E-07
TGM2	0.74	8.70E-10	1.92E-07
OLFML2A	-0.97	8.99E-10	1.95E-07
BCL2A1	2.28	1.02E-09	2.19E-07
TFCP2L1	-0.64	1.82E-09	3.83E-07
EDN1	1.08	5.90E-09	1.23E-06
TUBA1C	0.61	6.32E-09	1.29E-06
C18orf32	-2.11	1.64E-08	3.31E-06
TYMP	1.05	1.69E-08	3.37E-06
TSC22D3	-0.74	1.89E-08	3.68E-06
ALDH1A3	0.53	1.90E-08	3.68E-06
THBD	-0.98	2.02E-08	3.87E-06
CPA4	-0.62	2.25E-08	4.26E-06
KRT17	0.50	3.67E-08	6.84E-06
SAA2-SAA4	2.06	4.72E-08	8.69E-06
CLCA2	-0.59	5.83E-08	1.06E-05
GPSM3	4.69	6.73E-08	1.21E-05
KYNU	0.86	8.20E-08	1.46E-05
PI3	1.79	1.04E-07	1.82E-05
IFITM3	0.79	1.43E-07	2.48E-05
TUBA1A	0.57	1.59E-07	2.73E-05
XDH	0.74	1.76E-07	2.98E-05
PARP9	0.68	1.87E-07	3.11E-05
KRT4	1.05	1.88E-07	3.11E-05
NEDD9	0.64	2.27E-07	3.71E-05
MMP1	0.78	2.30E-07	3.72E-05
IFITM10	-1.80	2.33E-07	3.74E-05
FSBP	-1.50	2.55E-07	4.05E-05
MAST4	0.53	2.96E-07	4.63E-05
STAT1	0.52	3.15E-07	4.90E-05
DUSP4	0.65	3.45E-07	5.23E-05
HDGF	0.45	3.47E-07	5.23E-05
ADRB2	0.70	3.43E-07	5.23E-05
SERPINB2	0.71	3.88E-07	5.78E-05
CSTB	0.66	4.11E-07	6.06E-05
SLPI	0.58	5.38E-07	7.86E-05
STC1	0.74	5.67E-07	8.19E-05
PRODH	-2.20	6.83E-07	9.77E-05
S100P	0.80	8.08E-07	1.15E-04
FAM83A	0.49	9.18E-07	1.29E-04
MTSS1	0.57	9.33E-07	1.30E-04
GJB2	0.48	1.48E-06	2.03E-04
SOCS3	1.01	1.49E-06	2.03E-04
PLSCR1	0.83	1.65E-06	2.23E-04
PLAU	0.51	1.74E-06	2.33E-04
CCDC9B	0.62	1.78E-06	2.36E-04
ASH2L	-0.60	1.86E-06	2.44E-04
SGPP2	0.80	2.16E-06	2.82E-04
KCTD11	0.45	2.22E-06	2.87E-04
IFI44	0.82	2.28E-06	2.93E-04
VNN1	1.57	2.47E-06	3.13E-04
ALOX15B	0.56	2.48E-06	3.13E-04
FOSL1	0.57	2.57E-06	3.21E-04
ZBED2	0.63	2.80E-06	3.47E-04
LCN2	0.74	3.16E-06	3.88E-04
RHOV	0.64	3.30E-06	4.02E-04
SERPINB1	0.67	3.66E-06	4.38E-04
LYN	0.66	3.63E-06	4.38E-04
ITGA5	0.45	3.88E-06	4.61E-04
NANOS1	-1.99	3.95E-06	4.66E-04
SELENOP	-0.92	4.05E-06	4.74E-04
TGM1	0.71	4.18E-06	4.85E-04
MAF	-0.70	4.32E-06	4.97E-04
CXCL14	-1.34	4.60E-06	5.25E-04
VTCN1	-1.05	4.65E-06	5.27E-04
ENDOD1	-0.48	4.85E-06	5.46E-04
NFKB1	0.59	5.07E-06	5.66E-04
TMF1	-0.53	5.99E-06	6.64E-04
MYLK	-1.11	6.05E-06	6.66E-04
MAP7D2	-1.54	6.59E-06	7.20E-04
ANGPTL4	0.56	6.83E-06	7.40E-04
SESN3	-0.76	7.35E-06	7.91E-04
BCL3	0.79	7.45E-06	7.96E-04
PDGFB	0.87	7.62E-06	8.08E-04
IFIH1	0.74	8.79E-06	9.26E-04
LDLR	0.43	9.39E-06	9.82E-04
SPRR1B	0.81	1.07E-05	1.11E-03
DTX2	0.73	1.65E-05	1.70E-03
NID1	-1.30	1.77E-05	1.81E-03
CYP27B1	1.24	1.80E-05	1.82E-03
HELZ2	0.89	1.80E-05	1.82E-03
EFCAB7	-0.92	1.89E-05	1.90E-03
SLITRK6	-0.60	2.21E-05	2.20E-03
ZNF253	-1.13	2.51E-05	2.49E-03
DUSP10	-0.58	2.76E-05	2.71E-03
RAB7B	-0.50	2.95E-05	2.88E-03
FLOT1	-1.48	3.11E-05	3.01E-03
SERPINB13	-0.53	3.15E-05	3.04E-03
PIP4K2C	0.54	3.24E-05	3.10E-03
ZRANB1	-0.55	3.35E-05	3.19E-03
DRAM1	0.56	3.42E-05	3.23E-03
PPARGC1A	-1.20	3.45E-05	3.24E-03
FDPS	0.47	3.90E-05	3.64E-03
KRT23	0.61	3.99E-05	3.70E-03
CDRT1	1.14	4.10E-05	3.77E-03
TNFSF14	1.69	4.14E-05	3.79E-03
BMP2	0.77	4.20E-05	3.82E-03
ASS1	0.43	4.42E-05	3.95E-03
PDPN	0.47	4.41E-05	3.95E-03
KRT24	1.85	4.41E-05	3.95E-03
OTUB2	0.59	4.47E-05	3.97E-03
SLC25A37	0.62	4.67E-05	4.12E-03
SEMA7A	0.54	4.70E-05	4.13E-03
HSD11B1	0.55	4.89E-05	4.24E-03
MUC4	0.93	4.88E-05	4.24E-03
DEK	-0.51	5.47E-05	4.72E-03
PLAUR	0.58	6.32E-05	5.42E-03
TNC	0.40	6.39E-05	5.44E-03
USP9X	-0.57	6.44E-05	5.46E-03
TUBB3	0.68	6.50E-05	5.48E-03
EHD1	0.66	6.69E-05	5.60E-03
CLPB	0.59	6.80E-05	5.60E-03
SPTLC3	-0.78	6.79E-05	5.60E-03
HYI	0.56	6.74E-05	5.60E-03
NRDE2	-0.69	6.90E-05	5.66E-03
RWDD1	-0.71	7.31E-05	5.95E-03
PDCD4	-0.42	7.34E-05	5.95E-03
AC005154.5	-1.01	7.52E-05	6.06E-03
PFKFB3	0.49	7.83E-05	6.27E-03
ARPP19	-0.66	7.96E-05	6.35E-03
NEURL1B	0.49	8.19E-05	6.49E-03
HAS3	0.45	8.26E-05	6.51E-03
VPS52	1.35	8.44E-05	6.61E-03
CDCP1	0.37	8.64E-05	6.74E-03
FXYD5	0.41	8.75E-05	6.78E-03
CCDC14	-0.58	8.79E-05	6.78E-03
PRELID1	0.47	8.99E-05	6.90E-03
PTGS2	0.62	9.23E-05	7.05E-03
SLC9A7	0.55	9.71E-05	7.38E-03
NCCRP1	0.57	9.88E-05	7.47E-03
MX2	1.63	1.02E-04	7.68E-03
SULF2	-0.39	1.03E-04	7.68E-03
ANXA3	0.50	1.07E-04	7.92E-03
AL160269.1	-2.36	1.07E-04	7.92E-03
CCT5	0.41	1.13E-04	8.33E-03
ETS1	0.51	1.14E-04	8.35E-03
CDC42EP2	0.79	1.16E-04	8.37E-03
C1R	1.11	1.15E-04	8.37E-03
AC073610.3	2.17	1.15E-04	8.37E-03
FIGN	-1.28	1.19E-04	8.54E-03
TUBB	0.44	1.20E-04	8.61E-03
TUBB4B	0.42	1.22E-04	8.70E-03
RPL36A	-0.52	1.25E-04	8.84E-03
LRMP	-1.13	1.26E-04	8.89E-03
AL589880.1	-1.22	1.32E-04	9.26E-03
VEGFA	0.41	1.35E-04	9.40E-03
CD109	-0.45	1.35E-04	9.40E-03
PRSS23	-0.42	1.39E-04	9.63E-03
IDI1	0.40	1.41E-04	9.68E-03
F3	0.45	1.43E-04	9.82E-03
DDR1	0.40	1.44E-04	9.83E-03
IFNGR1	0.50	1.46E-04	9.89E-03

Supplementary Table 2

**Table:** Supplementary Table 2. Reporter metabolites.

Metabolites	P-values	Number of edges
Prenyl diphosphate(3-)	7.41E-05	2
Isocitrate(3-)	1.39E-04	1
Isopentenyl diphosphate(3-)	4.73E-04	3
Hydrogen peroxide	5.66E-04	8
L-proline	1.89E-03	2
Cysteaminium	2.09E-03	4
(4-hydroxyphenyl)acetaldehyde	2.28E-03	6
Farnesyl pyrophosphate(3-)	2.73E-03	1
Geranyl diphosphate(3-)	2.73E-03	1
3’-dephospho-CoA(2-)	2.77E-03	6
N2-Formyl-N1-(5-phospho-D-ribosyl)glycinamide	3.57E-03	2
N1-(5-Phospho-D-ribosyl)glycinamide	6.95E-03	1
L-phenylalanine	7.01E-03	10
GDP	7.96E-03	13
3,4-Dihydroxymandelate	8.38E-03	4
3,4-Dihydroxyphenylacetaldehyde	8.38E-03	4
(3,4-dihydroxyphenyl)acetate	8.38E-03	4
3-methoxy-4-hydroxyphenylglycolaldehyde	8.38E-03	4
3-Methoxy-4-hydroxymandelate	8.38E-03	4
4-hydroxyphenylacetate	8.38E-03	4
4-nitrophenolate	8.38E-03	4
4-nitrophenyl phosphate(2-)	8.38E-03	4
Glycochenodeoxycholate	8.38E-03	4
Nicotinate D-ribonucleotide	8.38E-03	4
D-ribitol	8.38E-03	4
Core 4	8.63E-03	5
(S)-2,3-epoxysqualene	8.84E-03	2
UDP-D-glucuronate	9.02E-03	5
Keratan sulfate I biosynthesis, precursor 36	9.49E-03	6
Keratan sulfate II biosynthesis, precursor 10	9.49E-03	6
Keratan sulfate II biosynthesis, precursor 10	9.49E-03	6
L-cysteine	9.90E-03	14
L-cysteine	1.03E-02	9
Pantetheine 4’-phosphate(2-)	1.04E-02	5
L-leucine	1.16E-02	10
Cholesta-5,7-dien-3beta-ol	1.18E-02	2
7-dehydrodesmosterol	1.18E-02	2
Uridine	1.23E-02	7
dCMP(2-)	1.27E-02	2
UDP(3-)	1.27E-02	2
UTP(4-)	1.27E-02	2
Supplementary Table 2. Continued.		
Metabolites	P-values	Number of edges
Deamino-NAD+	1.30E-02	5
Clupanodonyl CoA	1.37E-02	2
Gamma-linolenoyl-CoA(4-)	1.37E-02	2
Hydrogenphosphate	1.58E-02	74
{[galactosyl-(1,4)-N-Acetyl-glucosaminyl-(1,2)-mannosyl-(1,3)-],[N-acetyl-glucosaminyl-(1,2)-mannosyl-(1,6)-]mannosyl-(1,4)-N-acetyl-glucosaminyl-(1,4)-[fucosyl-(1,6)]-N-acetyl-glucosaminyl}asparagine	1.64E-02	5
3-methylimidazoleacetic acid	1.66E-02	5
D-ribulose 5-phosphate(2-)	1.73E-02	1
Diphosphate	1.81E-02	10
2-(formamido)-N1-(5-phospho-D-ribosyl)acetamidine	1.83E-02	2
Keratan sulfate I biosynthesis, precursor 11	1.86E-02	8
Keratan sulfate I biosynthesis, precursor 14	1.86E-02	8
Keratan sulfate I biosynthesis, precursor 17	1.86E-02	8
Keratan sulfate I biosynthesis, precursor 20	1.86E-02	8
Keratan sulfate I biosynthesis, precursor 23	1.86E-02	8
Keratan sulfate I biosynthesis, precursor 26	1.86E-02	8
Keratan sulfate I biosynthesis, precursor 29	1.86E-02	8
Keratan sulfate I biosynthesis, precursor 32	1.86E-02	8
Keratan sulfate I biosynthesis, precursor 35	1.86E-02	8
Keratan sulfate I biosynthesis, precursor 5	1.86E-02	8
Keratan sulfate I biosynthesis, precursor 8	1.86E-02	8
Keratan sulfate II biosynthesis, precursor 6	1.86E-02	8
Keratan sulfate II biosynthesis, precursor 9	1.86E-02	8
Keratan sulfate II biosynthesis, precursor 6	1.86E-02	8
Keratan sulfate II biosynthesis, precursor 9	1.86E-02	8
UDP-D-galactose(2-)	2.06E-02	9
Hydrogenphosphate	2.10E-02	30
Ethamp[c]	2.16E-02	5
Acetyl-CoA	2.19E-02	5
(R)-Pantothenate	2.20E-02	7
Keratan sulfate II biosynthesis, precursor 1	2.42E-02	7
Keratan sulfate II biosynthesis, precursor 1	2.42E-02	7
GTP	2.68E-02	12
Protoheme	2.81E-02	3
4,8 dimethylnonanoyl-CoA	2.84E-02	1
1-pyrroline-5-carboxylate	3.01E-02	3
ps_hs[m]	3.17E-02	3
5-phospho-beta-D-ribosylamine	3.52E-02	2
S-succinyldihydrolipoamide	3.84E-02	2
CDP(3-)	3.99E-02	1
CMP(2-)	3.99E-02	1
Supplementary Table 2. Continued.		
Metabolites	P-values	Number of edges
dCDP(3-)	3.99E-02	1
dCTP	3.99E-02	1
dCTP	3.99E-02	1
dGDP(2-)	3.99E-02	1
dGTP	3.99E-02	1
dGTP	3.99E-02	1
GDP(3-)	3.99E-02	1
GTP(4-)	3.99E-02	1
UMP(2-)	3.99E-02	1
N4-{N-acetyl-beta-D-glucosaminyl-(1,2)-alpha-D-mannosyl-(1,3)-[N-acetyl-beta-D-glucosaminyl-(1,2)-alpha-D-mannosyl-(1,6)]-beta-D-mannosyl-(1,4)-N-acetyl-beta-D-glucosaminyl-(1,4)-[alpha-L-fucosyl-(1,6)]-N-acetyl-beta-D-glucosaminyl}asparagine	4.03E-02	5
dGDP	4.26E-02	2
Pantetheine	4.28E-02	5
(6R)-5,10-methenyltetrahydrofolate	4.39E-02	6
Hexadecenoyl-CoA (n-C16:1CoA)	4.52E-02	2
Vaccenyl coenzyme A	4.52E-02	2
Apocarboxylase (Lys residue)	4.66E-02	1
Apocarboxylase (Lys residue)	4.66E-02	1
Holocarboxylase (biotin covalent bound to Lys residue of apoC)	4.66E-02	1
Holocarboxylase (biotin covalent bound to Lys residue of apoC)	4.66E-02	1
Biotinyl-5’-AMP	4.66E-02	1
Biotinyl-5’-AMP	4.66E-02	1
Dephospho-CoA	4.70E-02	5
L-leucine	4.74E-02	11
Globoside	4.94E-02	2
UMP	5.00E-02	1

Supplementary Table 3

**Table:** Supplementary Table 3. Reporter pathways.PathwaysP-valuesNumber of edgesNucleotide interconversion3.00E-09113Keratan sulfate synthesis8.15E-0770Keratan sulfate degradation4.97E-0676CoA synthesis2.02E-0434Fructose and mannose metabolism3.81E-0427Purine synthesis6.13E-0325Fatty acid synthesis1.16E-0254NAD metabolism1.61E-0223Squalene and cholesterol synthesis2.67E-026CoA catabolism3.33E-0214Phenylalanine metabolism3.79E-0220

Pathways	P-values	Number of edges
Nucleotide interconversion	3.00E-09	113
Keratan sulfate synthesis	8.15E-07	70
Keratan sulfate degradation	4.97E-06	76
CoA synthesis	2.02E-04	34
Fructose and mannose metabolism	3.81E-04	27
Purine synthesis	6.13E-03	25
Fatty acid synthesis	1.16E-02	54
NAD metabolism	1.61E-02	23
Squalene and cholesterol synthesis	2.67E-02	6
CoA catabolism	3.33E-02	14
Phenylalanine metabolism	3.79E-02	20

Supplementary Table 4

**Table:** Enrichment results of communities (KEGG).

Community	Term
1	JAK-STAT signalling pathway
1	EGFR tyrosine kinase inhibitor resistance
1	Cytokine-cytokine receptor interaction
1	Viral protein interaction with cytokine and cytokine receptor
1	Th17 cell differentiation
2	Complement and coagulation cascades
2	Fluid shear stress and atherosclerosis
2	Prostate cancer
2	Hematopoietic cell lineage
2	NF-kappa B signalling pathway
3	IL-17 signalling pathway
3	Viral protein interaction with cytokine and cytokine receptor
3	Chemokine signalling pathway
3	Rheumatoid arthritis
3	TNF signalling pathway
4	Measles
4	RIG-I-like receptor signalling pathway
4	Influenza A
4	NOD-like receptor signalling pathway
4	Epstein-Barr virus infection
5	Gap junction
5	Phagosome
5	Pathogenic Escherichia coli infection
5	Huntington disease
5	Alzheimer disease
6	NF-kappa B signalling pathway
6	C-type lectin receptor signalling pathway
6	Osteoclast differentiation
6	Apoptosis
6	Epstein-Barr virus infection
7	Focal adhesion
7	PI3K-Akt signalling pathway
7	ECM-receptor interaction
7	Human papillomavirus infection
7	Fluid shear stress and atherosclerosis
8	TNF signalling pathway
8	IL-17 signalling pathway
8	NF-kappa B signalling pathway
8	Cytokine-cytokine receptor interaction
8	Transcriptional misregulation in cancer
9	C-type lectin receptor signalling pathway
9	Kaposi sarcoma-associated herpesvirus infection
Supplementary Table 4. Continued.	
Community	Term
9	Leishmaniasis
9	Osteoclast differentiation
9	Necroptosis
10	TGF-beta signalling pathway
10	Signalling pathways regulating pluripotency of stem cells
10	Staphylococcus aureus infection
10	Cytokine-cytokine receptor interaction
10	Tuberculosis

Supplementary Table 5

**Table:** Enrichment results of communities (GO-BP).

Community	Term
1	Positive regulation of tyrosine phosphorylation of STAT protein
1	Regulation of tyrosine phosphorylation of STAT protein
1	Tyrosine phosphorylation of STAT protein
1	Positive regulation of JAK-STAT cascade
1	Positive regulation of STAT cascade
2	Regulation of wound healing
2	Regulation of response to wounding
2	Regulation of blood coagulation
2	Regulation of hemostasis
2	Regulation of coagulation
3	Chemokine-mediated signalling pathway
3	Response to chemokine
3	Cellular response to chemokine
3	Neutrophil chemotaxis
3	Neutrophil migration
4	Negative regulation of type I interferon production
4	Activation of innate immune response
4	Positive regulation of innate immune response
4	Pattern recognition receptor signalling pathway
4	Regulation of innate immune response
5	Sequestering of metal ion
5	Ciliary basal body-plasma membrane docking
5	Organelle localization by membrane tethering
5	Membrane docking
5	Regulation of G2/M transition of mitotic cell cycle
6	Regulation of type I interferon production
6	Type I interferon production
6	Regulation of intrinsic apoptotic signalling pathway
6	NIK/NF-kappa B signalling
6	Intrinsic apoptotic signalling pathway
Supplementary Table 5. Continued.	
Community	Term
7	Positive chemotaxis
7	Vascular endothelial growth factor receptor signalling pathway
7	Extracellular matrix organization
7	Positive regulation of peptidyl-tyrosine phosphorylation
7	Extracellular structure organization
8	Cellular response to tumour necrosis factor
8	Response to tumour necrosis factor
8	Regulation of endopeptidase activity
8	Cellular response to lipopolysaccharide
8	T cell migration
9	Type I interferon signalling pathway
9	Cellular response to type I interferon
9	Response to type I interferon
9	Response to virus
9	Response to interferon-gamma
10	Activin receptor signalling pathway
10	Positive regulation of pathway-restricted SMAD protein phosphorylation
10	Regulation of pathway-restricted SMAD protein phosphorylation
10	Pathway-restricted SMAD protein phosphorylation
10	Regulation of transmembrane receptor protein serine/threonine kinase signalling pathway

Supplementary Figure 1ACE2 expression.
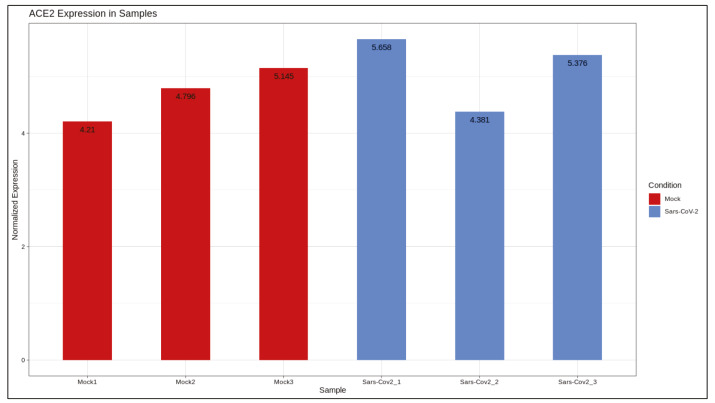

Supplementary Figure 2Dotplot of enrichment results of upregulated genes (KEGG).
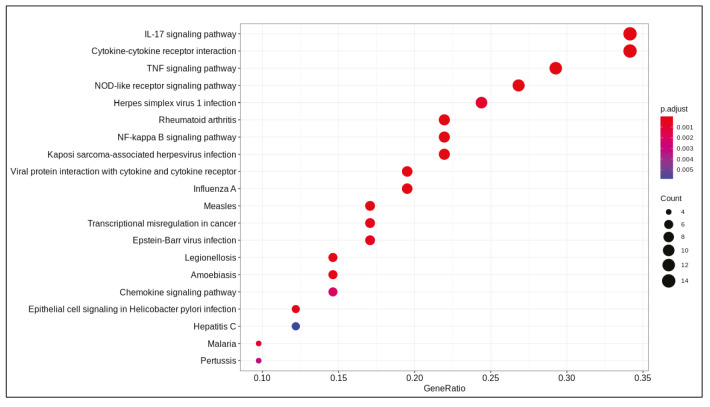

Supplementary Figure 3Dotplot of enrichment results of downregulated genes (KEGG).
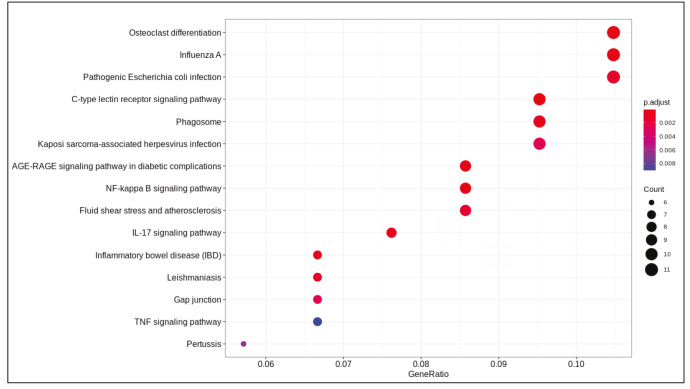

Supplementary Figure 4Dotplot of enrichment results of upregulated genes (GO-BP).
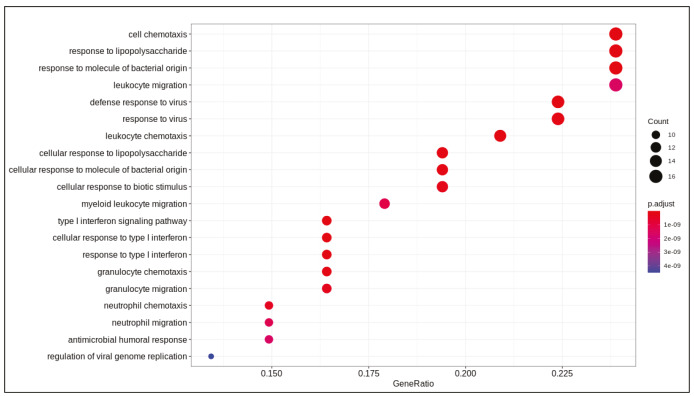

Supplementary Figure 5Dotplot of enrichment results of downregulated genes (GO-BP).
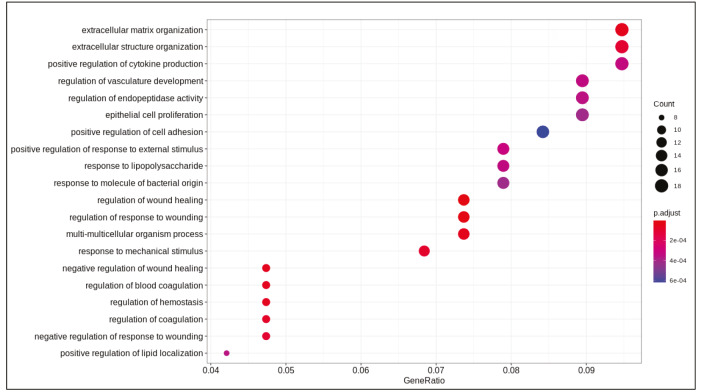

Supplementary Figure 6Heatmap of enrichment results of communities (KEGG).
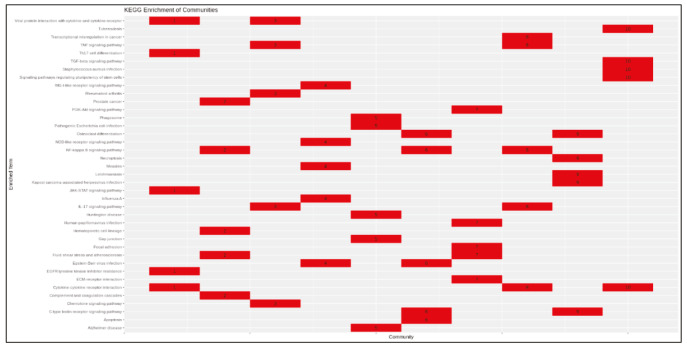

Supplementary Figure 7Heatmap of enrichment results of communities (GO-BP).
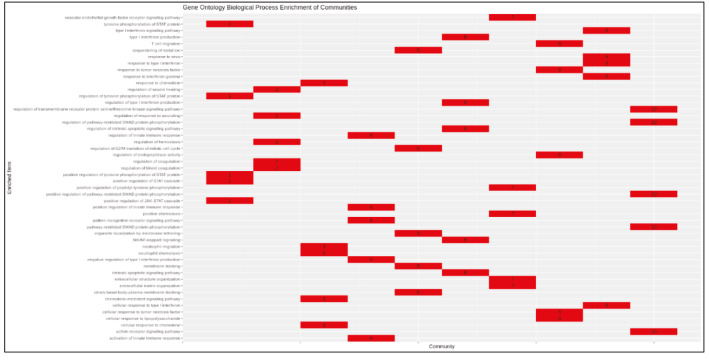

Supplementary Figure 8Clustering pattern of protein-protein interaction network (ClusterONE).
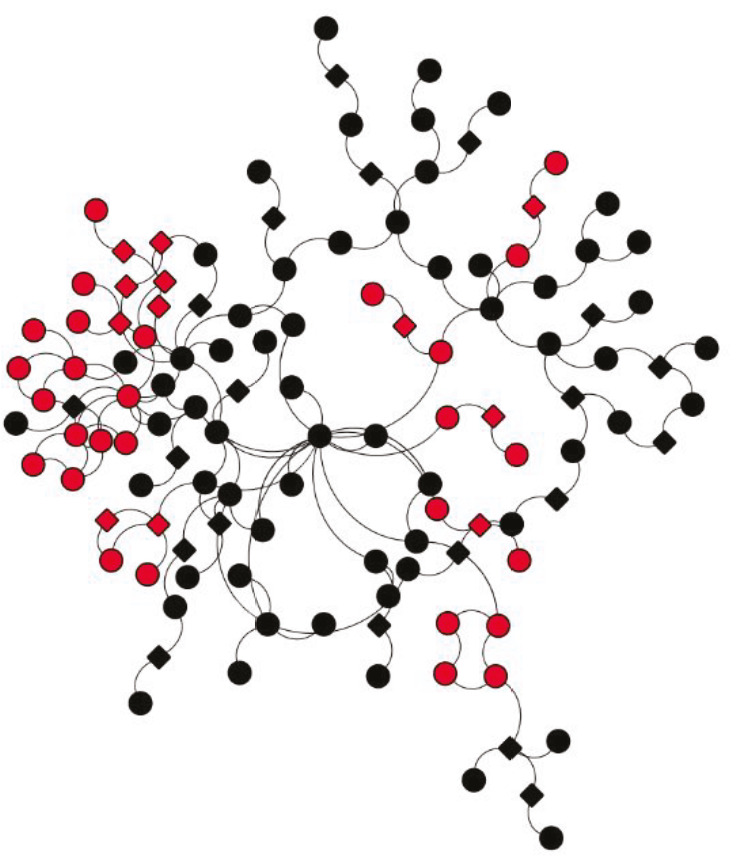

Supplementary Figure 9Heatmap of enrichment results of protein clusters (KEGG).
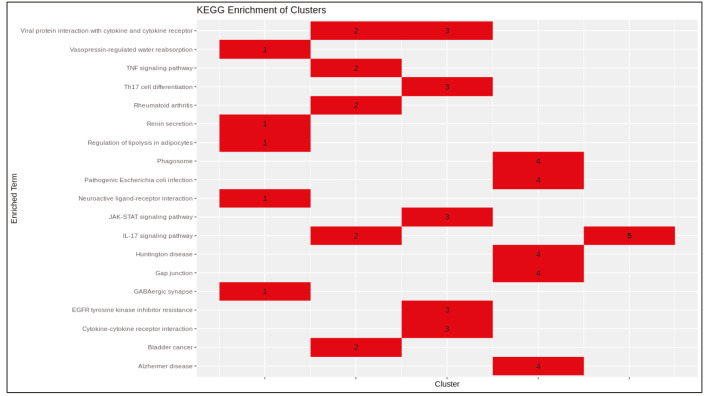

Supplementary Figure 10Heatmap of enrichment results of protein clusters (GO-BP).
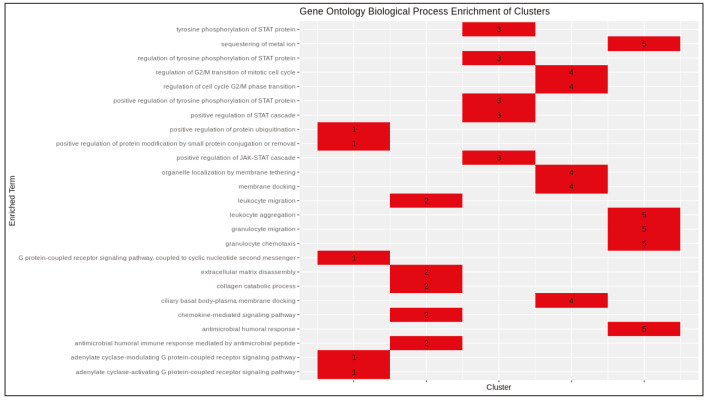

Supplementary Figure 11Communities of clustered protein modules.